# Correction: The cross-sectional association of stressful life events with depression severity among patients with hypertension and diabetes in Malawi

**DOI:** 10.1371/journal.pone.0304887

**Published:** 2024-05-31

**Authors:** Kelsey R. Landrum, Brian W. Pence, Bradley N. Gaynes, Josée M. Dussault, Mina C. Hosseinipour, Kazione Kulisewa, Jullita Kenela Malava, Jones Masiye, Harriet Akello, Michael Udedi, Chifundo C. Zimba

The S3 File is uploaded incorrectly. The authors have provided correct version of S3 File below.

## Supporting information

S3 File (PDF)
