# Supplementary material for: The cross-sectional association of stressful life events with depression severity among patients with hypertension and diabetes in Malawi
Source: PLoS One. 2022 Dec 30;17(12):e0279619. doi: 10.1371/journal.pone.0279619 (PMC9803137; doi:10.1371/journal.pone.0279619)
Supplement: S1 File — (PDF) [file pone.0279619.s001.pdf]

SF 1. PHQ-9 Survey (PDF)

SECTION C. DEPRESSION AND ANXIETY SYMPTOMS

Source: PHQ-9 + GAD-7

The next set of questions ask how you have been feeling **over the past two weeks**.

During the past two weeks, how many days have you been bothered by feeling down, depressed, or hopeless?	0	0 days
	1	1-7 days
	2	8-12 days
	3	13 or 14 days
During the past two weeks, how many days have you been bothered by little interest or pleasure in doing things?	0	0 days
	1	1-7 days
	2	8-12 days
	3	13 or 14 days
During the past two weeks, how many days have you been bothered by trouble falling or staying asleep, or sleeping too much?	0	0 days
	1	1-7 days
	2	8-12 days
	3	13 or 14 days
During the past two weeks, how many days have you been bothered by feeling tired, fatigued or having little energy?	0	0 days
	1	1-7 days
	2	8-12 days
	3	13 or 14 days
During the past two weeks, how many days have you been bothered by poor appetite or overeating?	0	0 days
	1	1-7 days
	2	8-12 days
	3	13 or 14 days
During the past two weeks, how many days have you been bothered by feeling bad about yourself—or that you are a failure or have let yourself or your family down?	0	0 days
	1	1-7 days
	2	8-12 days
	3	13 or 14 days
During the past two weeks, how many days have you been bothered by trouble concentrating on things, such as reading, talking with friends, finishing tasks, or attending a meeting?	0	0 days
	1	1-7 days
	2	8-12 days
	3	13 or 14 days
During the past two weeks, how many days have you been bothered by moving or speaking so slowly that	0	0 days
	1	1-7 days

other people could have noticed? Or the opposite—being so fidgety or restless that you have been moving around a lot more than usual?	2	8-12 days
	3	13 or 14 days
During the past two weeks, how many days have you been bothered by thoughts that you would be better off dead or of hurting yourself in some way?	0	0 days
	1	1-7 days
	2	8-12 days
	3	13 or 14 days
**STOP: IF ANSWER TO QUESTION 9 IS >0, COMPLETE SUICIDE RISK ASSESSMENT PROTOCOL AND INDICATE ASSESSMENT RESULT:**	Suicide assessment result is:<br>☐ **Passive**<br>☐ **Active: Low risk**<br>☐ **Active: Moderate/high risk**<br>☐ **Active: Acute risk**	
