# Supplementary material for: The cross-sectional association of stressful life events with depression severity among patients with hypertension and diabetes in Malawi
Source: PLoS One. 2022 Dec 30;17(12):e0279619. doi: 10.1371/journal.pone.0279619 (PMC9803137; doi:10.1371/journal.pone.0279619)
Supplement: S2 File — (PDF) [file pone.0279619.s002.pdf]

SF 2. Stressful life events survey (PDF).

SECTION D. STRESSFUL LIFE EVENTS		
Source: LES (CHASE Study)		
Many people go through difficult times. The next few questions ask about stressful events you may have experienced **in the past 3 months.**		
**In the last 3 months**, have you had any significant relationship changes, such as:		
Getting married or engaged **in the last 3 months**?	1<br>2	Yes<br>No
Having an increase in serious arguments with your spouse or boyfriend/girlfriend **in the last 3 months**?	1<br>2	Yes<br>No
Getting divorced or separated **in the last 3 months**?	1<br>2	Yes<br>No
Having a major change in closeness to a family member, such as estrangement from a family member **in the last 3 months**?	1<br>2	Yes<br>No
**In the last 3 months**, have you experienced the death of a close family member such as a:		
Husband, wife, or partner **in the last 3 months**?	1<br>2	Yes<br>No
Child **in the last 3 months**?	1<br>2	Yes<br>No
Mother **in the last 3 months**?	1<br>2	Yes<br>No
Father **in the last 3 months**?	1<br>2	Yes<br>No
Brother or sister **in the last 3 months**?	1<br>2	Yes<br>No
Grandparent **in the last 3 months**?	1<br>2	Yes<br>No
Other **in the last 3 months (if yes, please explain)**?	1<br>2	Yes<br>No
You chose "other" please explain which close family member passed away in the last 3 months		
**In the past 3 months**, have you experienced the death of a very close friend?	1<br>2	Yes<br>No
**In the past 3 months**, have you experienced a serious illness or injury of any close family members? Please do not include those whom you've mentioned that died.		
Husband, wife, or partner **in the last 3 months?**	1	Yes

	2	No
Child **<u>in the last 3 months?</u>**	1<br>2	Yes<br>No
Mother **<u>in the last 3 months?</u>**	1<br>2	Yes<br>No
Father **<u>in the last 3 months?</u>**	1<br>2	Yes<br>No
Brother or sister **<u>in the last 3 months?</u>**	1<br>2	Yes<br>No
Grandparent **<u>in the last 3 months?</u>**	1<br>2	Yes<br>No
Other **<u>in the last 3 months (if yes, please explain)</u>**?	1<br>2	Yes<br>No
You chose "other" please explain which close family member passed away in the last 3 months		
**<u>In the past 3 months</u>**, have you experienced a serious illness or injury of a very close friend? Please do not include those whom you've mentioned that died.	1<br>2	Yes<br>No
**<u>In the past 3 months</u>**, have you experienced any significant work-related difficulties such as:		
Being unable to find work even though you were looking for it **<u>in the last 3 months?</u>**	1<br>2	Yes<br>No
Losing your work or source of income, or being in danger of losing your work or source of income **<u>in the last 3 months?</u>**	1<br>2	Yes<br>No
Having trouble with your employer such as being suspended or demoted, experiencing discrimination, or any other major problems with your job **<u>in the last 3 months?</u>**	1<br>2	Yes<br>No
**<u>In the past 3 months</u>**, have you had a major new illness, injury, or health problem?	1<br>2	Yes<br>No
**<u>In the past 3 months</u>**, have you been hospitalized?	1<br>2	Yes<br>No
**<u>In the past 3 months</u>**, have you been in a motor vehicle accident? Don't include minor accidents with no injury or significant damage.	1<br>2	Yes<br>No
**<u>In the past 3 months</u>**, were you physically attacked or assaulted or had your life threatened?	1<br>2	Yes<br>No

**<u>In the past 3 months</u>**, were you robbed or was your home burglarized?	1<br>2	Yes<br>No
**<u>In the past 3 months</u>**, have you felt unsafe in your neighborhood?	1<br>2	Yes<br>No

Included in analysis as an SLE:

M12	In the past month, how many days were you hungry when you went to sleep at night because there was not enough food?	__ __	Number of days<br>Don't know 98
